# Facile Fabrication of BiOI/BiOCl Immobilized Films With Improved Visible Light Photocatalytic Performance

**DOI:** 10.3389/fchem.2018.00058

**Published:** 2018-03-12

**Authors:** Yingxian Zhong, Yuehua Liu, Shuang Wu, Yi Zhu, Hongbin Chen, Xiang Yu, Yuanming Zhang

**Affiliations:** ^1^Department of Chemistry, Jinan University, Guangzhou, China; ^2^Analytical and Testing Center, Jinan University, Guangzhou, China

**Keywords:** BiOI/BiOCl film, visible light, heterojunction, photodegradation, recycle

## Abstract

**HIGHLIGHTS**

- A facial method was used to fabricate BiOI/BiOCl film at room temperature.
- 30% BiOI/BiOCl showed an excellent photocatalytic activity and stability.
- Improvement of photocatalytic activity was owed to expanded visible light absorption and high separation efficiency of charge.

Photocatalysis has been considered to be one of the most promising ways to photodegrade organic pollutants. Herein, a series of BiOI/BiOCl films coating on FTO were fabricated through a simple method at room temperature. The photocatalytic efficiency of 30%BiOI/BiOCl could reach more than 99% aiming to degrading RhB and MB after 90 and 120 min, respectively. Compared with BiOCl, 30%BiOI/BiOCl showed 12 times higher efficiency when degrading RhB. In comparison with BiOI, 30%BiOI/BiOCl showed 5 and 6 times higher efficiency when degrading RhB and MB, respectively. These obvious enhancements were attributed to expanded visible light absorption and high separation performance of photoinduced charge. Moreover, the photocatalytic activity of 30%BiOI/BiOCl had no obvious decrease after five recycles, suggesting that it was a promising photocatalyst for the removal of MB and RhB pollutants. Finally, the possible growth process for the BiOI/BiOCl thin films and photocatalysis mechanism were investigated in details. This work would provide insight to the reasonable construction of BiOX heterojunction and the photocatalytic mechanism in degrading organic pollutants.

## Introduction

Recently, semiconductor photocatalysts have been potential materials in energy storage, organic pollutants degradation and so on Kisch (2013). Since TiO_2_ had been reported to produce H_2_ under UV light (Fujishima and Honda, 1972), transitional mental oxides have been applied as photocatalysts, such as ZnO (Soci et al., 2007), SnO_2_ (Law et al., 2002), and WO_3_ (Baeck et al., 2003). However, many of them have wide bandgap and are activated by UV-light (4% of solar light). To utilize more solar light, searchers pay a lot of efforts to find new photocatalysts which could maximize the utilization of solar light. Among those photocatalysts, BiOCl is considered as a new kind of promising layered material for photocatalysis due to its unique layered structure, high chemical and optical stability, corrosion resistance and nontoxicity (Li J. et al., 2014; Ding et al., 2015; Li et al., 2017). BiOCl has layered structure consisting of [Bi_2_O_2_]^2+^ sandwiched between two slabs of Cl^−^, which produces internal static electric fields to separate photogenerated electrons and holes (Cheng et al., 2014; Mi et al., 2016). However, the practical application of BiOCl has been hindered owing to its wide bandgap and relatively high recombination rate of photoinduced carriers (Dong et al., 2012; Xiao et al., 2012).

Aiming at solving these shortcomings, many strategies have been reported to enhance the photocatalytic efficiency of BiOCl, including: (i) impurity element doping, such as BiOCl_x_Br_y_I_z_ (Sun X. et al., 2015) and BiOCl_x_I_1−x_ (Kim et al., 2014), (ii) surface functionalization, like inducing oxygen vacancies in BiOCl (Jiang et al., 2013), (iii) construction of the plasmonic photocatalysis system, such as Ag/BiOCl (Liu H. et al., 2012) and Ag-AgX-BiOX (X = Cl, Br, I) (Cheng et al., 2011; Xiong et al., 2011; Cao et al., 2013), (iv) construction of semiconductor heterojunctions (Jiang et al., 2011; Wang et al., 2015). Construction of semiconductor heterojunctions has been widely explored in recent years because of two advantages. First, materials with wide bandgap could match with lots of semiconductors at the energy level. In that way, it is propitious to electron and hole separation by building an interfacial electric field between different semiconductors. Cui's work showed that photodegradation efficiency of Ag_3_PO_4_/BiOI was nearly 10 times that of BiOI (Cui et al., 2013). And Cui's group found that photodegradation efficiency of BiOI/Bi_2_WO_6_ was about 6.1 times higher than that of pure Bi_2_WO_6_ under visible light irradiation (Li et al., 2013). Ao's work showed that Ag_2_MoO_4_/g-C_3_N_4_ highly improved photocatalytic degradation performance for different organic pollutants under sunlight irradiation (Wu et al., in press). Secondly, coupled with narrow band semiconductors, BiOCl could expand visible light absorption and utilize more solar energy. Narrow bandgap materials act as the light absorber and generate photoinduced carriers with proper energy, indicating that it is a very efficient visible-light-activated photocatalyst (Wang et al., 2017). Therefore, many BiOCl/narrow bandgap materials, such as BiOCl-C_3_N_4_ (Wang et al., 2013), BiOCl/Bi_24_O_31_Cl_10_ (Li F. et al., 2014), BiOCl/Bi_2_S_3_ (Cheng et al., 2012), BiOCl/BiOI (Sun L. et al., 2015), BiOCl/BiOBr (Zhang et al., 2013), and NaBiO_3_/BiOCl (Chang et al., 2010), have been successfully prepared.

Based on the advantages mentioned above, BiOI is a great candidate to couple with BiOCl, which is a narrow bandgap semiconductor (1.72 eV) and has a similar layered structure (Jiang et al., 2011; Huang et al., 2015; Ning et al., 2016). Once coupled with BiOI, BiOI/BiOCl is expected to achieve the aims as followed: (i) to enhance visible light absorption, (ii) to accelerate separation efficiency of photoinduced electrons and holes (Cao et al., 2011; Xiao et al., 2012; Wang et al., 2016). Although there are a few reports about BiOI/BiOCl, most of them are powder synthesized through hydrothermal and solvothermal methods, which needs high temperature and pressure. Additionally, powder catalysts are hard to be separated and recovered because they are easily dispersed into solution when used in pollutants degradation (Zhao et al., 2015). Unlike powder catalysts, immobilized photocatalysts become more promising in practical application for easy separation and high reusability (Liu X. et al., 2012). Therefore, BiOI/BiOCl film is of great advantage in practical organic pollutants degradation.

In this work, a facial method was used to fabricate a series of BiOI/BiOCl immobilized films at room temperature. The possible growth process of BiOI/BiOCl film was investigated in detail. All BiOI/BiOCl films showed better photocatalytic performance than pristine BiOCl film. UV-vis diffusion reflectance spectra, photocurrent, fluorescence spectra (PL) and trapping experiment were used to gain insights into the reasons for remarkable enhancement of photocatalytic activity and the possible photocatalysis mechanism of BiOI/BiOCl film. Besides, recycle experiments were used to measure the stability and duration of BiOI/BiOCl film.

## Experimental

### Synthesis of *x*BiOI/BiOCl film

**Scheme 1:**
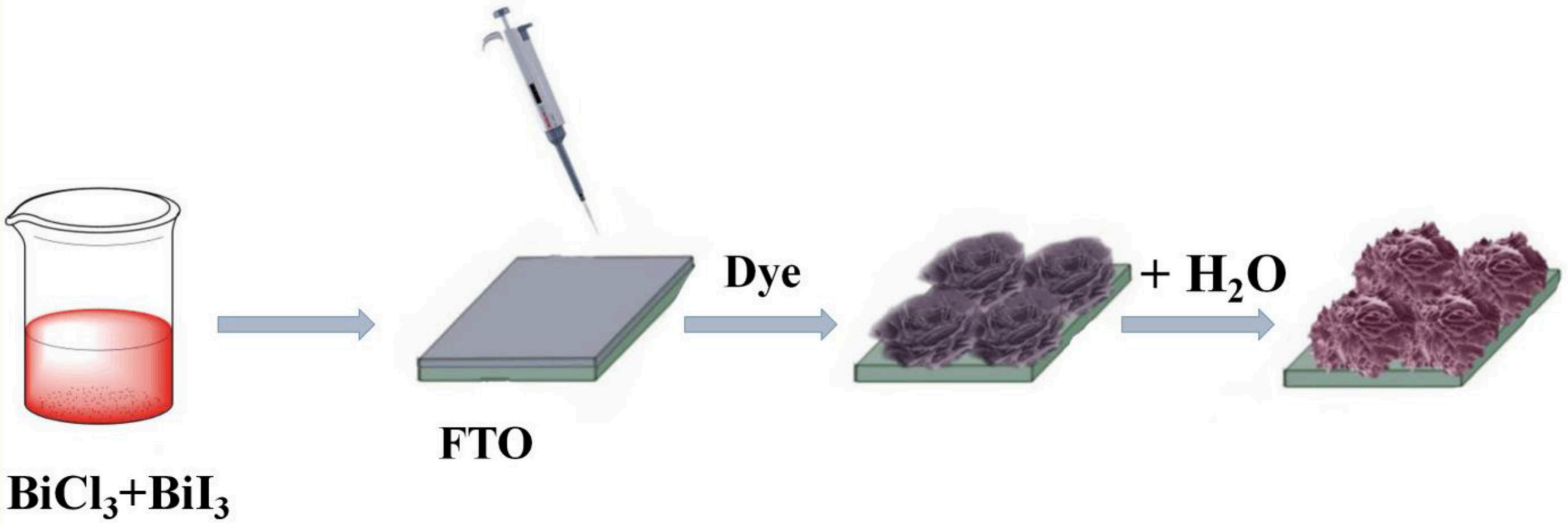
Illustration of the preparation of BiOI/BiOCl composites on FTO.

In a typical procedure, 3.0 g BiCl_3_ was mixed with 100 mL ethanol and 1 mL HCl, and stirred for 1 h to form BiCl_3_ solution. Similarly, BiI_3_ solution was prepared using BiI_3_, HI and ethanol in the same way. After that, BiCl_3_ solution and BiI_3_ solution were mixed with different molar ratio.1 mL of mixture solution was dropped onto FTO glass. After being dried at 100°C for 1 h, the films were dipped into distilled water for 30 min to form BiOI/BiOCl (as shown in Scheme 1). Finally, the samples dried at 60°C for 2 h. The *x*BiOI/BiOCl composites with molar ratios of BiOI to BiOCl at 10, 30, and 60% were named as 10%BiOI/BiOCl, 30%BiOI/BiOCl, 60%BiOI/BiOCl, respectively.

### Characterization of photocatalysts

The morphologies and phase structures of *x*BiOI/BiOCl films were observed by Field emission scanning electron microscopy (FE-SEM, Zeiss ULTRA 55), transmission electron microscopy (JEOL 2010F) and high-resolution transmission electron microscopy (JEOL 2100 F) and X-ray diffractometry (XRD, equipped with a Cu Ka X-ray source). The optical properties of as-synthesized catalysts were tested by UV–vis spectrophotometer (DRS, Hitachi- UV-3010, using BaSO_4_ for the baseline measurement) and photoluminescence spectroscopy (PL, RF-5301PC). FT-IR spectra were recorded on an Aipha-Centuart FT-IR spectrometer.

The visible-light-driven photocatalytic efficiencies of *x*BiOI/BiOCl films were evaluated the degradation of Rhodamine B (RhB, 2.5 mg L^−1^) and methylene blue (MB, 2.5 mg L^−1^) in a reactor equipped with a 350 W Xe lamp with >420 nm filter as the light source. The as-obtained BiOI/BiOCl film was putting into a reactor, in which 100 mL dye solution were poured. Before irradiation, the solution was continuously stirring in the dark for 30 min to ensure establish adsorption-desorption equilibrium. At certain time interval, 4 mL of the suspension were sampled; the concentration of dye solution was measured by recording the absorption band maximum in the absorption spectra. For comparison, the photocatalytic activities of BiOCl and BiOI were characterized under same condition. In addition, 30%BiOI/BiOCl photocatalyst was examined by 5-cycle to characterize its stability. Before entering next cycle, samples were washed by deionized water and alcohol three times. Dried at 100°C for 1 h and reuse in fresh dye solution.

### Electrochemical measurements

Photocurrent of samples was studied by there-electrode system in a quartz cell, which was using Pt plate as counter electrode, Ag/AgCl as reference electrode, and the as-prepared samples as working electrode on electrochmical workstation (CHI660C.Shang-hai.). 0.1 M Na_2_SO_4_ solution was used as the electrolyte. The surface area of the working electrode was 4 × 5 cm^2^. A 350 W Xe lamp with an filter (λ > 420 nm) was used as the visible-light source.

## Results and discussion

### XRD patterns

**Figure 1:**
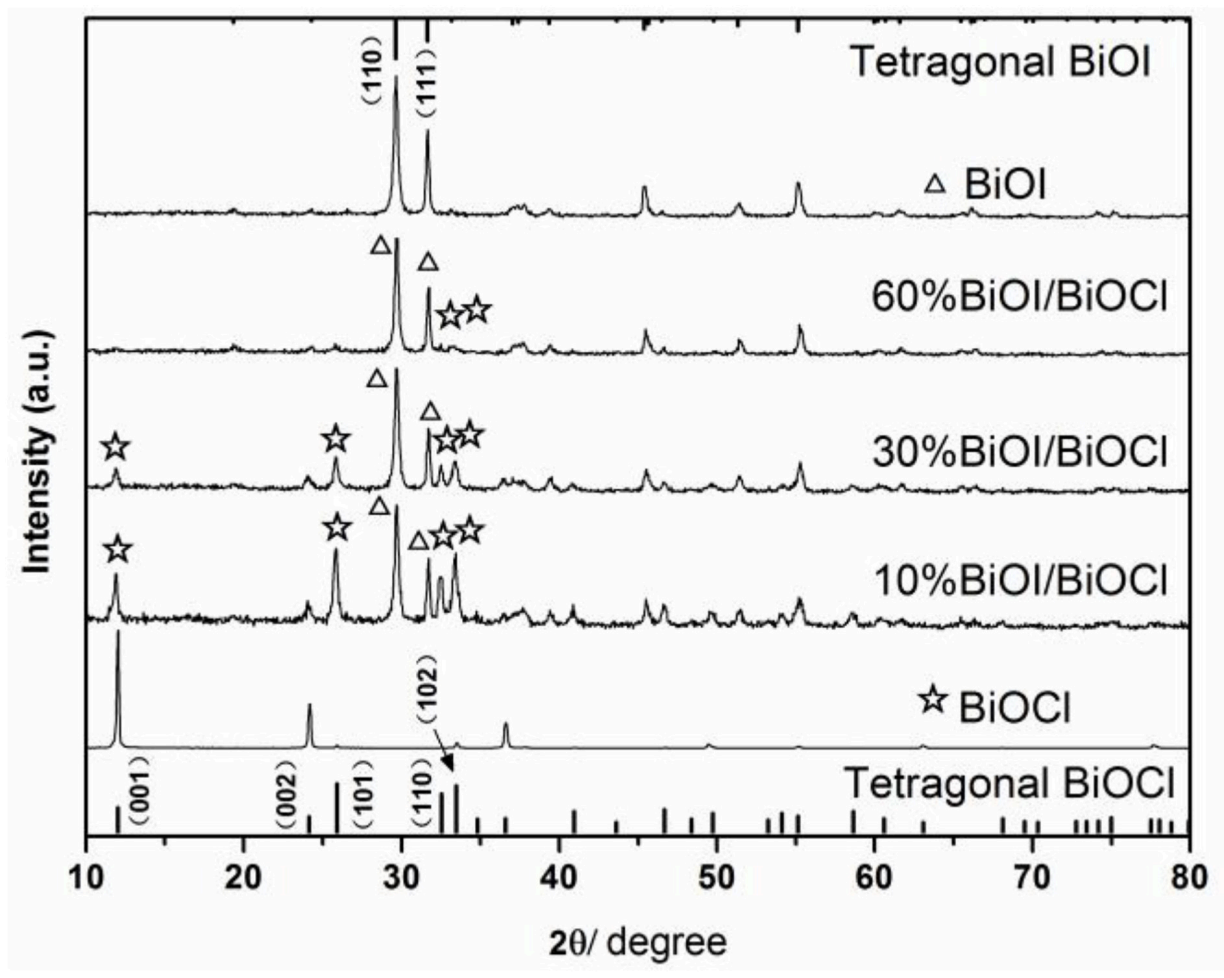
XRD patterns of the as-prepared films.

Figure 1 showed XRD patterns of the as-prepared *x*BiOI/BiOCl films. It could be seen that all the diffraction peaks of BiOI and BiOCl were in good agreement with the standard cards (JCPDS No. 73-2062) and (JCPDS No. 06-0249) without any impurity peaks, which indicated that they exhibited tetragonal structure and corresponded to the FT-IR results (Figure S2). The characteristic peaks of BiOI and BiOCl coexisted in the XRD patterns, demonstrating the formation of BiOI/BiOCl composite without the present of BiOCl_*x*_I_1−*x*_ solid solutions (Huang et al., 2015). With the increase of percentage of BiOI in the composites, the strength of diffraction peaks of BiOI gradually increased, on the contrary, the intensity of BiOCl simultaneously decreased. Additional, it could see that FTO peaks in Figure 1, it might be due to the uneven film on glass of the sample.

### SEM, TEM, HRTEM, and EDS images

**Figure 2:**
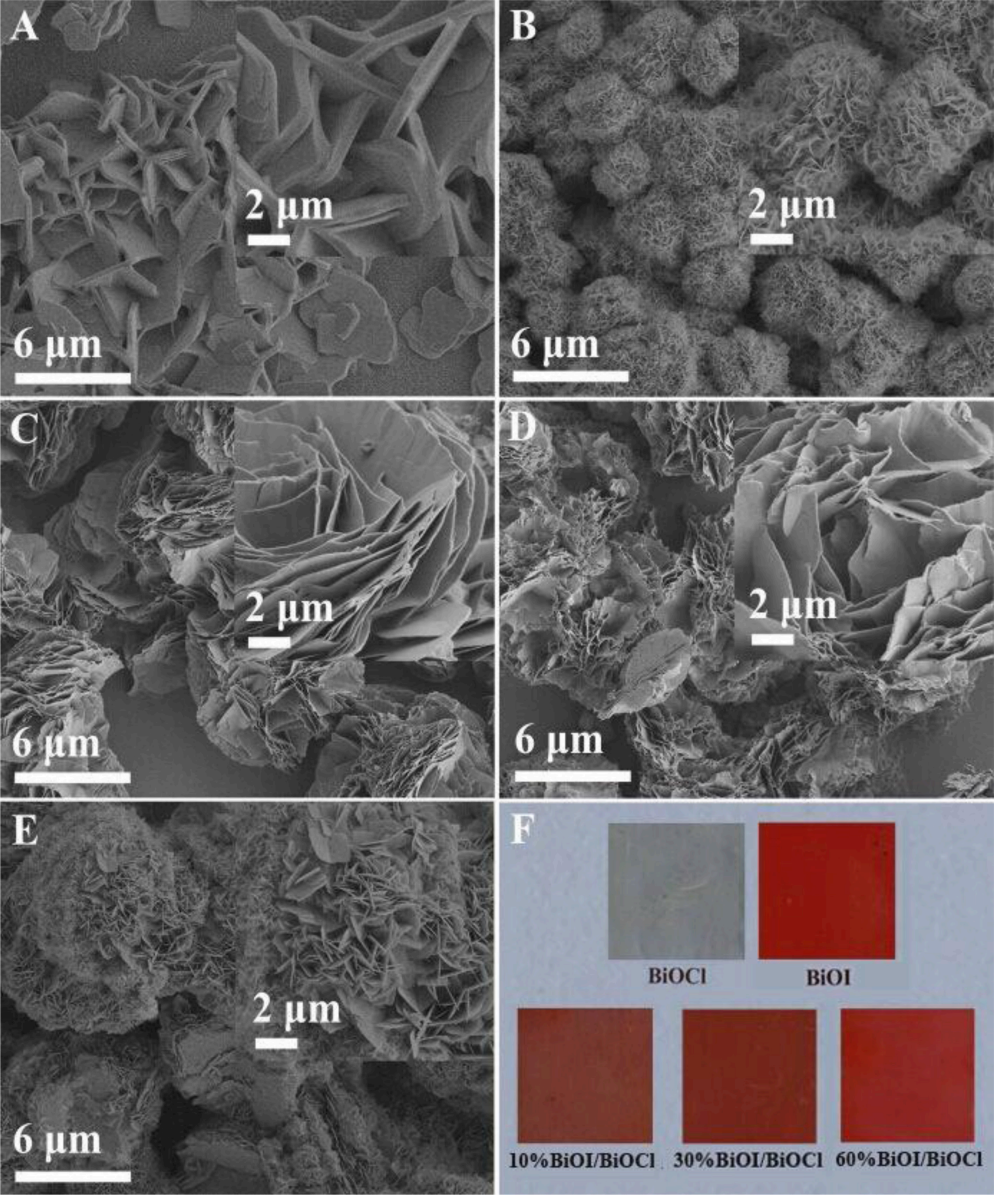
SEM images of BiOCl, BiOI and BiOI/BiOCl film: **(A)** BiOCl;**(B)** BiOI; **(C)** 10%BiOI/BiOCl; **(D)** 30%BiOI/BiOCl; **(E)** 60%BiOI/BiOCl; **(F)** Picture of as-synthesized samples.

All the samples were systematically analyzed by SEM. From Figure 2A, it could be observed that pristine BiOCl was composed of numerous nanosheets and its surface was very smooth. Differently, under similar preparation conditions, pristine BiOI presented hierarchical microspheres consisting of a series nanosheet in Figure 2B. As for *x*BiOI/BiOCl (Figures 2C–E), it could be observed that *x*BiOI/BiOCl showed hierarchical structure with BiOCl nanosheets adhering tightly on BiOI and the particle sizes of *x*BiOI/BiOCl obviously increased in comparison with pristine BiOCl. Additionally, color of sample gradually deepened compared with pristine BiOCl when percentage of BiOI increased in Figure 2F.

**Figure 3:**
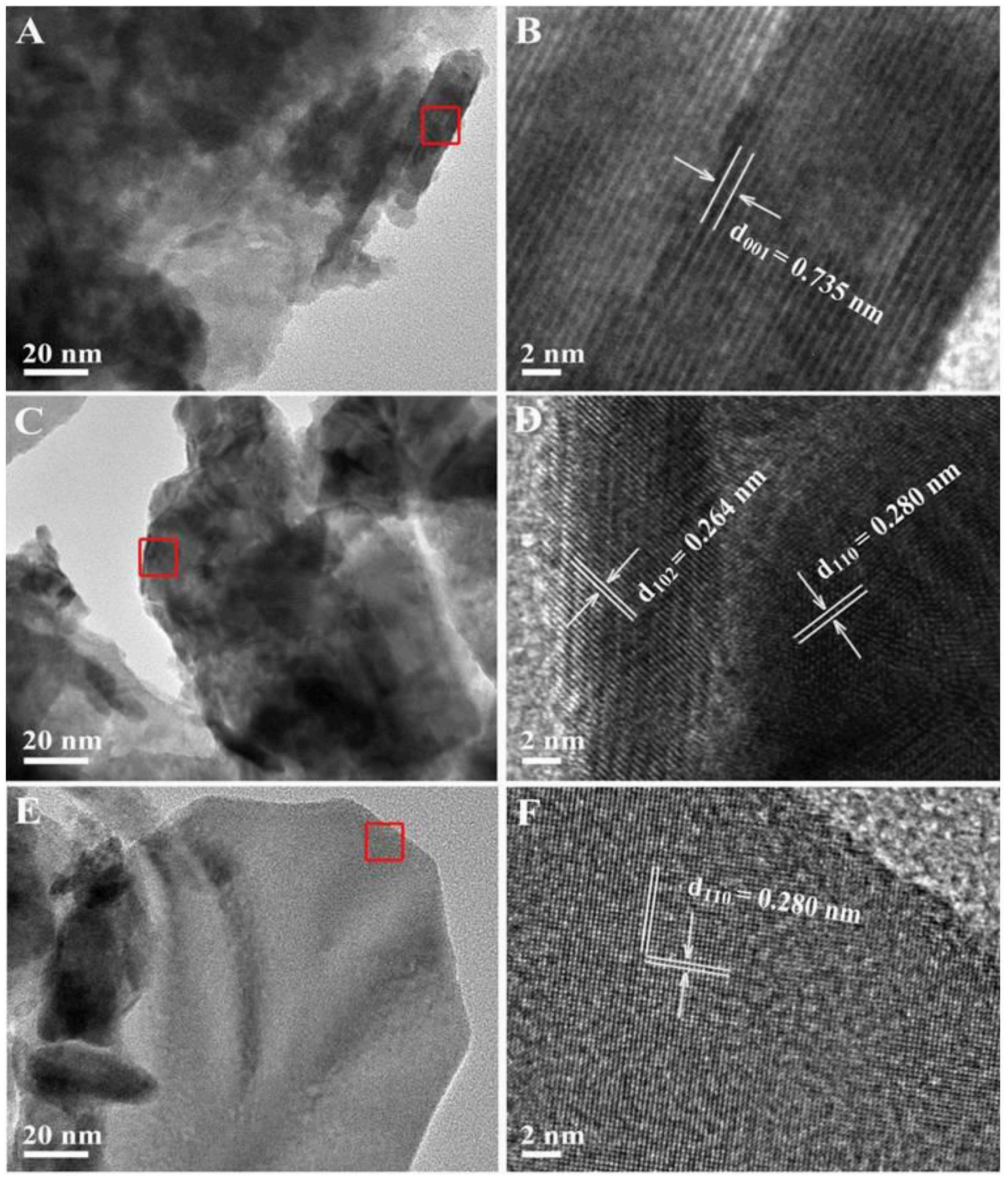
**(A)** TEM and **(B)** HRTEM images of BiOCl, **(C)** TEM and **(D)** HRTEM of 30%BiOI/BiOCl, **(E)** TEM and **(F)** HRTEM of BiOI.

**Figure 4:**
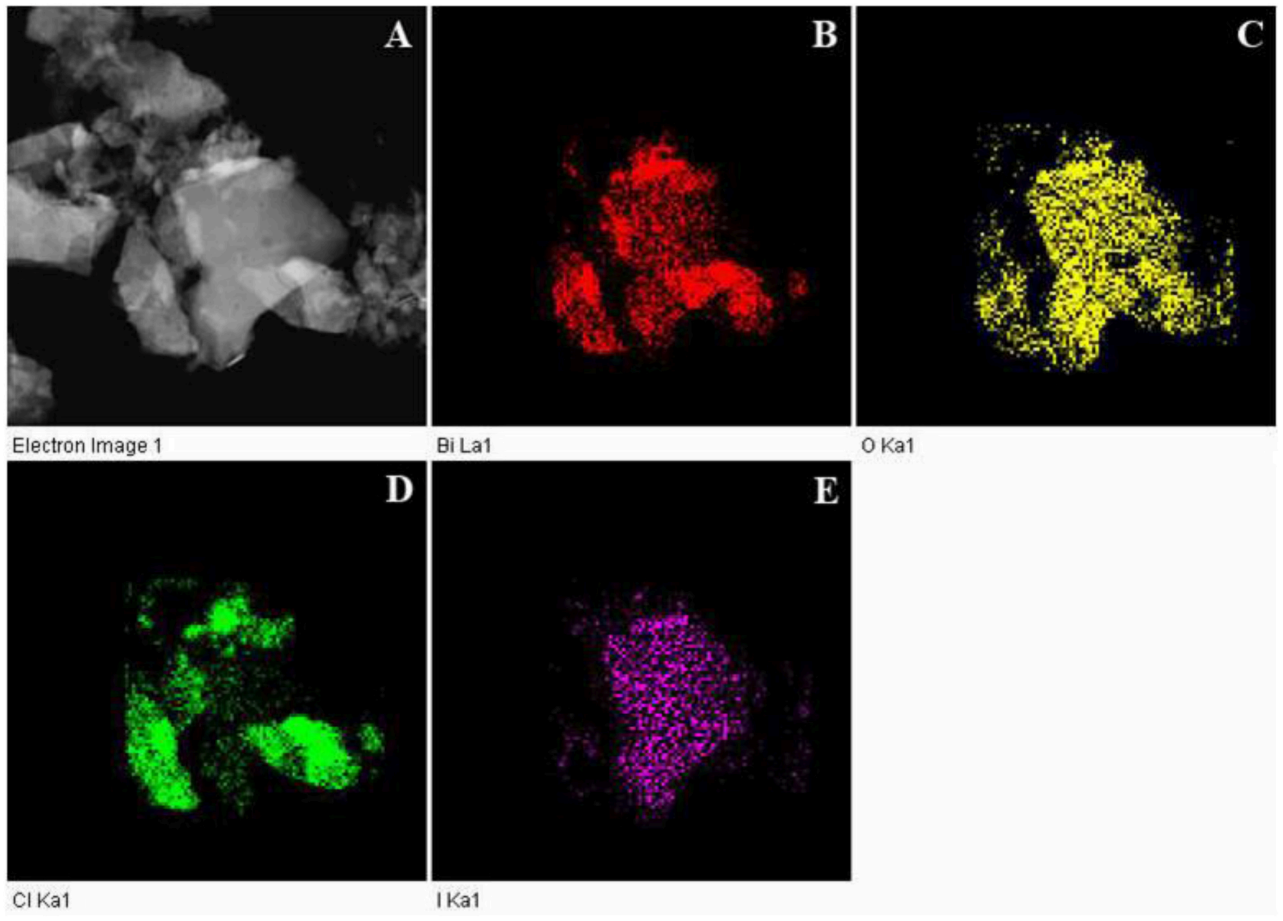
EDS images of 30%BiOI/BiOCl **(A)** and Bi **(B)**, O **(C)**, Cl **(D)**, I **(E)** elemental maps.

The morphology and structure of as-obtained samples were further characterized by TEM and HRTEM images. The microstructures of pristine BiOCl, pristine BiOI and 30%BiOI/BiOCl were shown in Figure 3. The interactions between BiOCl and BiOI were so strong that ultrasonication did not separate them during the sample preparation procedure for TEM characterization (Xiao and Zhang, 2010). Figures 3B,D,F indicated that the samples were highly crystallized. In Figure 3B, the lattice fringe with a *d*-spacing of 0.735 nm matched well with (001) lattice plane of BiOCl, while in Figure 3F, the interlayer distance of 0.280 nm responsed to the (280) plane of BiOI. Figure 3D showed the HRTEM of 30%BiOI/BiOCl, clear fringes with the lattice spacing of 0.264 and 0.280 nm could be indexed to (102) lattice plane of BiOCl and (110) lattice plane of BiOI, respectively. TEM results were in good consistent with XRD patterns in Figure 1. The results clearly confirmed the formation of heterostructure between BiOCl and BiOI. In addition, the elemental distributions of 30%BiOI/BiOCl were studied through EDS elemental mapping. The corresponding results for 30%BiOI/BiOCl were shown in Figures 4A–E. It could be obviously seen that the sample consist of only I, Bi, Cl, O, elements. The results of EDS mapping confirmed the composition, structure and the high purity of 30% BiOI/BiOCl composite.

### Growth process of *x*BiOI/BiOCl film

**Figure 5:**
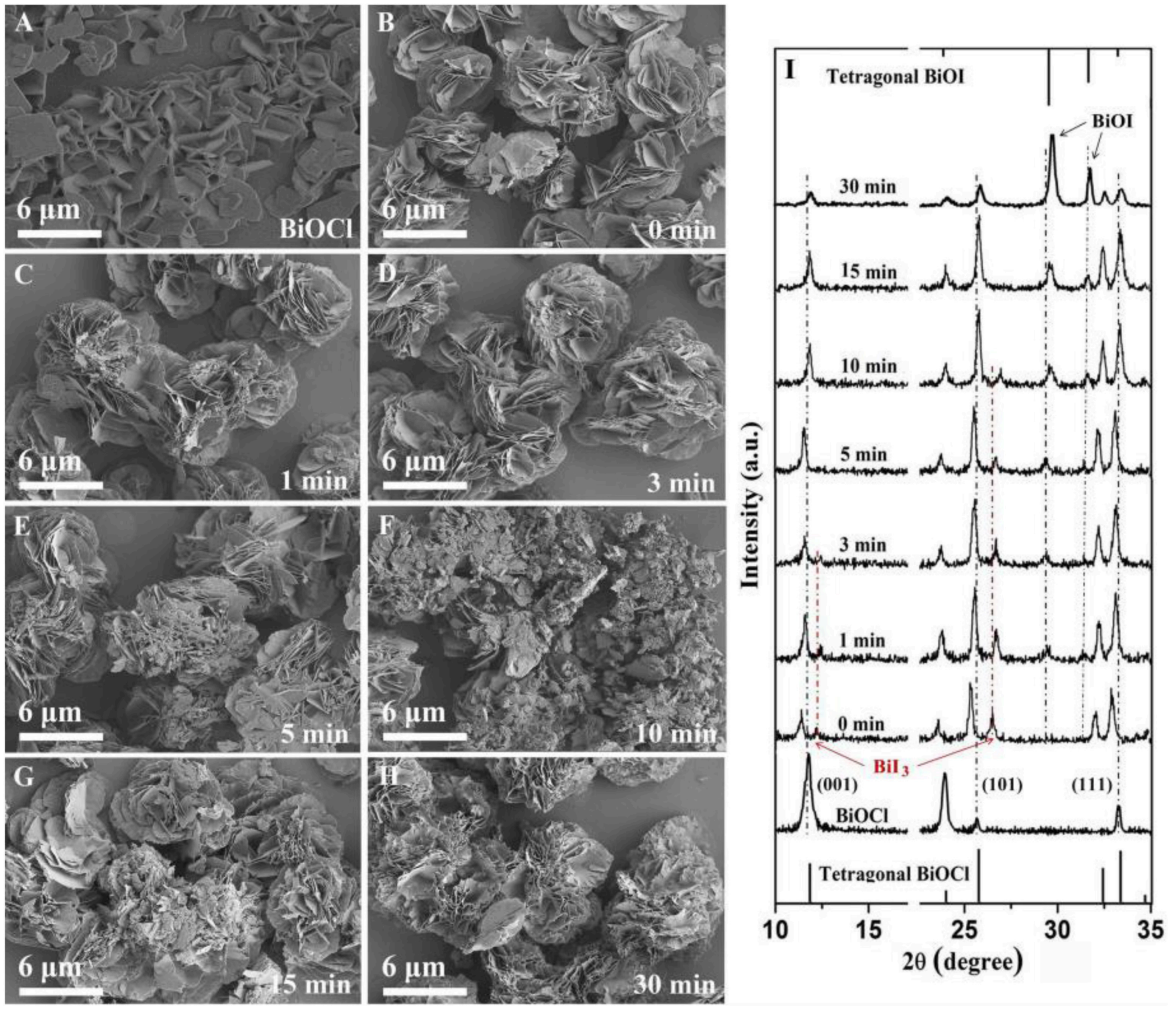
SEM images of **(A)** BiOCl and 30%BiOI/BiOCl after different hydrolysis times **(B)** 0 min, **(C)** 1 min, **(D)** 3 min, **(E)** 5 min, **(F)** 10 min, **(G)** 15 min, **(H)** 30 min, **(I)** XRD patterns of 30%BiOI/BiOCl after different hydrolysis time.

In order to understand the growth process of BiOI/BiOCl film in this work, SEM images and XRD patterns of 30%BiOI/BiOCl film at different time stage were shown in Figure 5. The whole process was divided into three stages. In the first stage (0 min), as shown in Figure 5I, the peaks collected from 0 min sample could be indexed as a composition of BiOCl and BiI_3_. After the mix solution consisting of BiI_3_ and BiCl_3_ was dropped on FTO, there was a hydrolyzation competition between them. BiCl_3_ was hydrolyzed to form BiOCl prior to the hydrolyzation of BiI_3_ when ethanol volatilized, because the K_sp_ (BiOCl) was smaller than K_sp_ (BiOI). As shown in Figure 5B, BiI_3_ broke down on the nanosheets structure of BiOCl to form into hierarchical structure. Besides, the diffraction peak at around 11.9° corresponding to the (001) plane shifted to smaller 2θ in Figure 5I. That might be because the ionic radius of I^−^ (220 pm) was larger than that of Cl^−^ (181 pm). In the second stage (1–15 min), BiI_3_ was hydrolyzed to BiOI. In Figure 5I, with the increase of reaction time, diffraction peak of BiI_3_ disappeared and the intensity of BiOI became stronger. In the meantime, the extent of hydrolyzation caused the shifting of the diffraction peak of (001) to bigger 2θ. Figures 5C–E showed that hierarchical BiOI and nanosheets-structure BiOCl formed a tidily hierarchical structure in the second stage. In the third stage (30 min), BiI_3_ was hydrolyzed totally, and BiOI/BiOCl was formed.

### Optical properties

**Figure 6:**
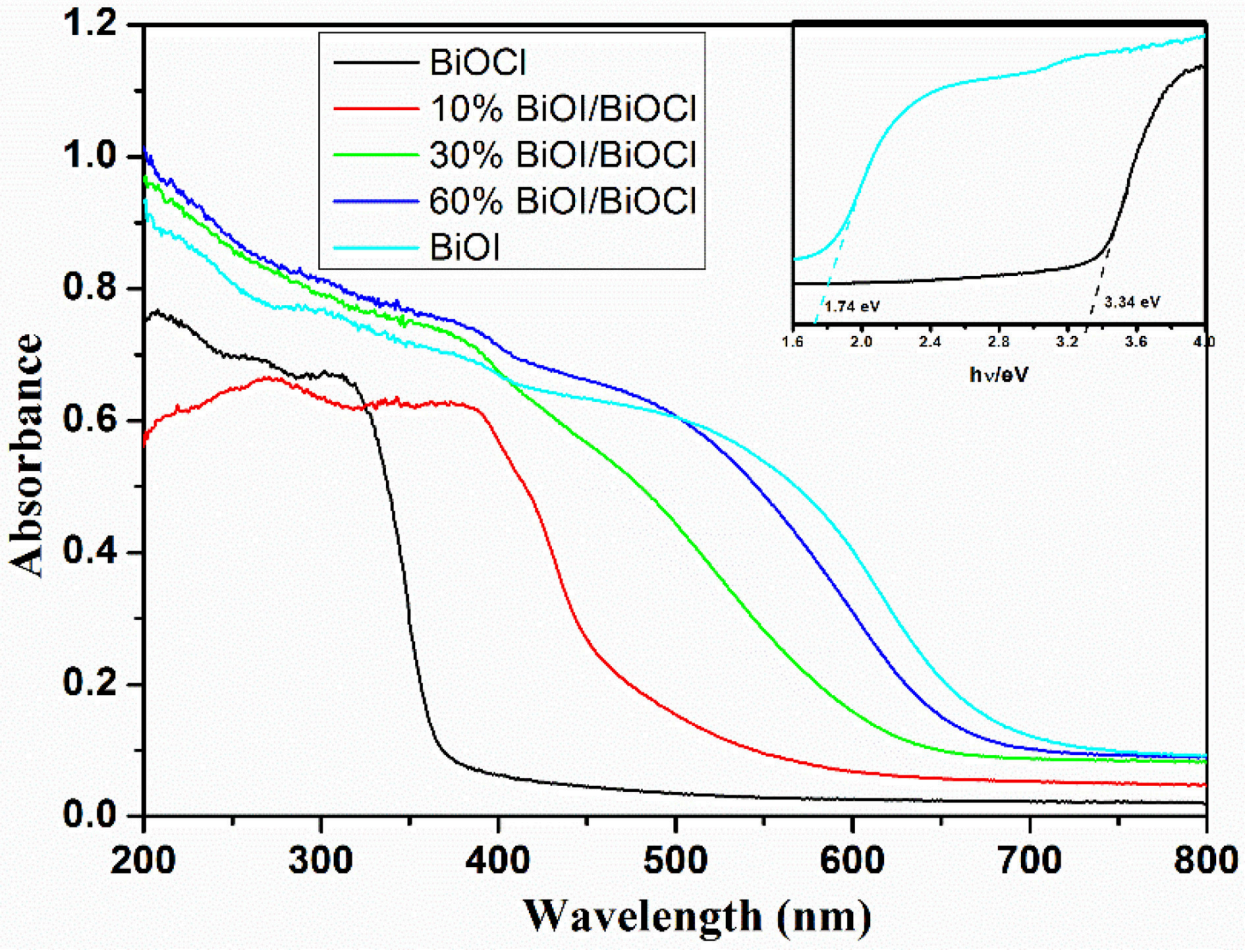
UV-vis diffuse reflectance spectrum (DRS) of *x*BiOI/BiOCl films.

The UV-vis diffuse reflectance spectra (DRS) of xBiOI/BiOCl films were shown in Figure 6. BiOCl had a strong absorption edge around 360 nm, meanwhile, BiOI had a strong absorption edge around 700 nm. Compared to BiOCl, *x*BiOI/BiOCl showed an absorption edge shifting to larger wavelength with the increase of BiOI percentage. This shifting was in accordance with the color change of as-prepared samples (Figure 2F) caused by the addition of BiOI. The band gap energy of a semiconductor could be calculated from the following equation:

(1) $$\begin{array}{r} \alpha h\nu =A{\left(h\nu -Eg\right)}^{n/2} \end{array}$$

where α, ν, *Eg*, and *A* were the absorption coefficient, light frequency, band gap energy, and a constant, respectively (Ning et al., 2016). Among them, *n* depended on the characteristics of the transition in a semiconductor. For example, *n* = 1 (direct transition) or *n* = 4 (indirect transition). BiOX belonged to indirection transition, thus *n* was estimated to be 4. The band gap of BiOI and BiOCl were 1.74 and 3.34 eV, respectively. With narrowing of band gap, *x*BiOI/BiOCl could exhibit enhanced visible light absorption, subsequently resulting in improved photocatalytic activity.

**Figure 7:**
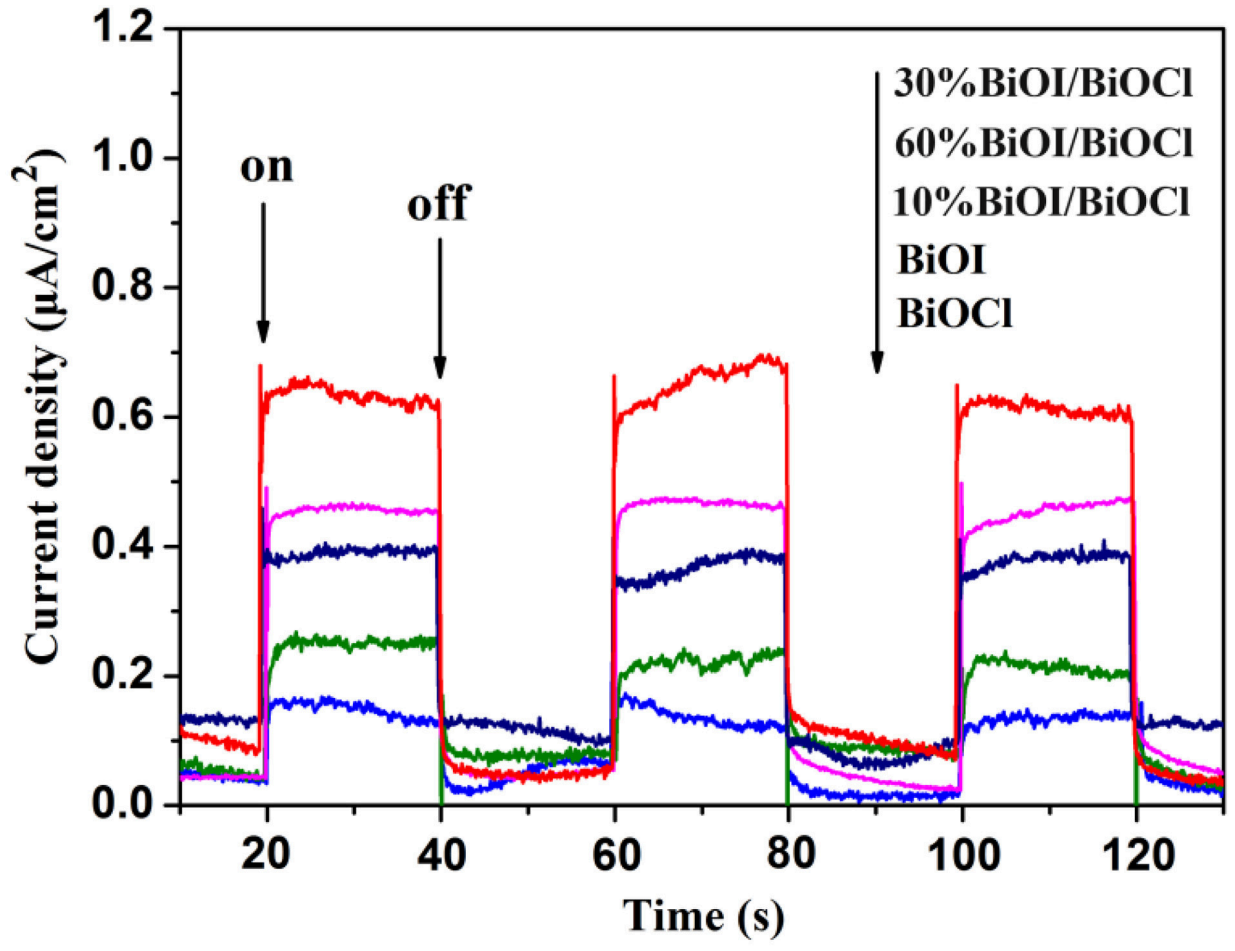
Visible light photocurrent of *x*BiOI/BiOCl films.

Besides optical absorption property, separation efficiency of photogenerated carriers played an important role in photodegradation. Photocurrent could directly indicate the capability of charge separation. The higher photocurrent density corresponded to the greater capability of charge separation. Figure 7 showed the photocurrent responses of the as-prepared photocatalysts under several on/off visible light irradiation cycles. BiOCl and BiOI showed a poor photocurrent response while the response of *x*BiOI/BiOCl increased. The photocurrent density of 30 %BiOI/BiOCl was almost 6 times as high as that of pristine BiOCl and 3 times as high as that of pristine BiOI, revealing that 30 %BiOI/BiOCl had superior separation efficiency of photogenerated carriers.

**Figure 8:**
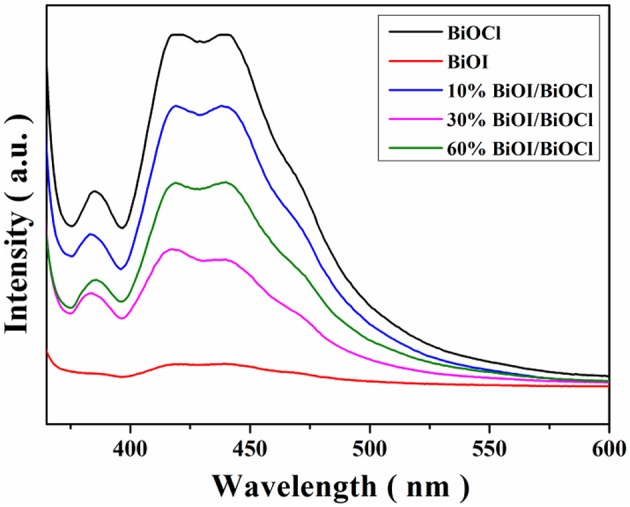
Fluorescence spectra of *x*BiOI/BiOCl films.

Photoluminescence spectra were used to characterize the photogenerated carriers' recombination rate of as-prepared samples, since the PL emission originated from free carrier's recombination. The higher PL intensity meaned the higher recombination rate in the photocatalytic procedure (Cao et al., 2011). As shown in Figure 8, BiOCl showed a strong emission peak with high intensity at approximate 420 and 440 nm, meanwhile, BiOI exhibited a low intensity. Decline of the PL intensity implied that adding BiOI could successful suppress recombination process during photocatalysis. In addition, 30%BiOI/BiOCl shown the lower intensity indicated the lower recombination rate, thus could promote photocatalytic activity.

### Photocatalytic properties

**Figure 9:**
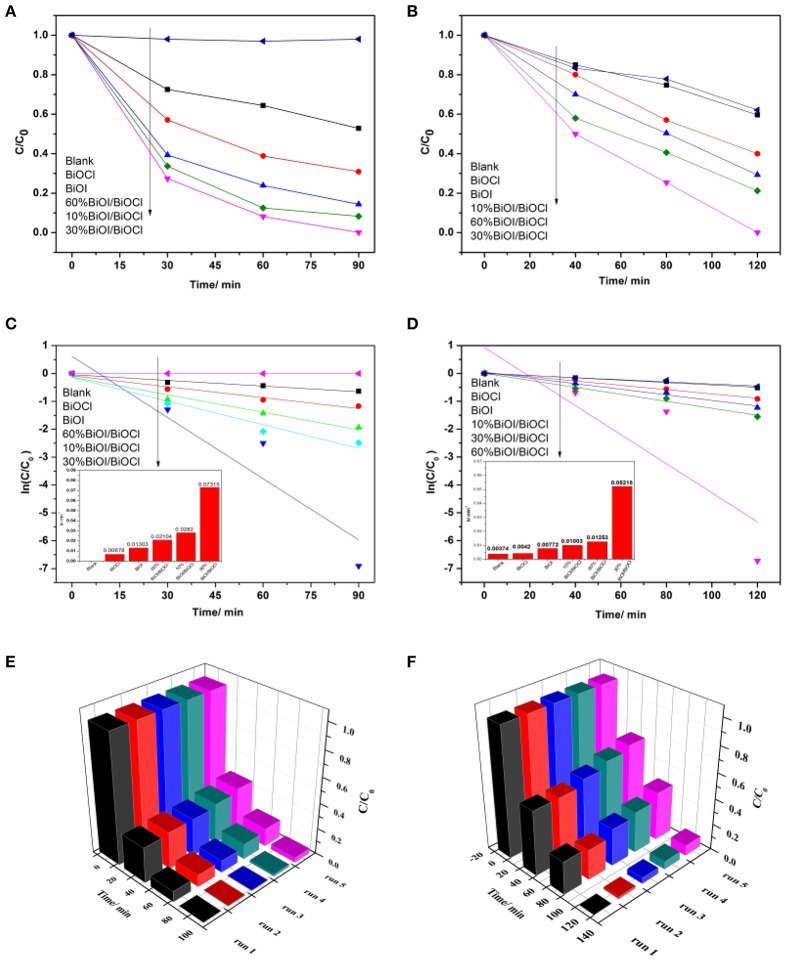
Photodegradation of dyes using *x*BiOI/BiOCl films: **(A)** RhB; **(B)** MB; pseudo-first-order reaction kinetics of 30%BiOI/BiOCl film: **(C)** RhB; **(D)** MB; and repeated degradation of dyes with 30%BiOI/BiOCl film under visible light irradiation: **(E)** RhB; **(F)** MB.

The photodegradation efficiency of the *x*BiOI/BiOCl films were evaluated by degradating RhB and MB under visible-light irradiation. As shown in Figure 9A, the degradation percentage of RhB by pristine BiOCl was 48% in 90 min. It was about 70% by pristine BiOI in 90 min. Compared with pristine BiOCl and BiOI, *x*BiOI/BiOCl film showed a great degradation: 30% BiOI/BiOCl could degrade more than 99% of RhB in 90 min. To further illustrate the photocatalytic reaction, pseudo-first-order kinetics were fitted from the degradation process (Ning et al., 2016),

(2) $$\begin{array}{r} \text{ln}\left({\text{C}}_{0}/\text{C}\right)=k\text{t} \end{array}$$

where the value of rate constant *k* was equal to the corresponding slope of the fitting line as shown in Figure 9C. The rate constant value for 30%BiOI/BiOCl was 0.07315 min^−1^, which was 12 times higher than BiOCl (0.00575 min^−1^) and 5 times higher than BiOI (0.01303 min^−1^), respectively. Figure 9B showed the photocatalytic performance of the *x*BiOI/BiOCl evaluated by degradating MB under visible-light irradiation. 48% of MB was self-degraded under visible light irradiation. Compared with self-degradation of MB, the photocatalytic performance of BiOCl was negligible and BiOI could only degrade 60% MB. The photocatalytic performance of *x*BiOI/BiOCl film was much better than pristine BiOCl and BiOI. More than 99% of MB was degraded using 30%BiOI/BiOCl film in 120 min. According to Figure 9D, the rate constant value of 30%BiOI/BiOCl was 0.05218 min^−1^, which was 6 times higher than BiOI (0.00772 min^−1^). This better photocatalytic performance might be due to the enhanced visible light absorption and improved separation efficiency of photoinduced carriers.

### Reusability of 30%BiOI/BiOCl film

Efforts were made in this work to identify the stability and practicality of as prepared catalysts for dye degradation, which was a significant factor to be considered in real application. 30%BiOI/BiOCl film was reused for RhB and MB degradation in five cycles under the same condition and the result was shown in Figures 9E,F. It was remarkable that the efficiencies had no obvious decrease after 5 cycles, revealing its great reusability. In term of XRD patterns in Figure S1, there was no obvious change in phase and structure of 30%BiOI/BiOCl film after 5 cycles, demonstrating its excellent stability. The excellent reusability and stability indicated its great potential in practical application.

### Photocatalytic mechanism

The energy band structures of BiOX were evaluated using the following equation (Xiao et al., 2016):

(3) $$\begin{array}{rcl} {\text{E}}_{\text{VB}} & = & \text{X}-{\text{E}}^{\text{e}}+{0.5\text{E}}_{\text{g}} \end{array}$$

(4) $$\begin{array}{rcl} {\text{E}}_{\text{CB}} & = & {\text{E}}_{\text{VB}}-{\text{E}}_{\text{g}} \end{array}$$

Where E_VB_ was the valence band edge potentials, X was the electronegativity of BiOX, which was the geometric mean of the electronegativity of constituent atoms, E^e^ was the energy of free electrons on the hydrogen scale (about 4.5 eV), E_g_ was the band gap energy, E_CB_ was the conductance band edge potentials (Xiao et al., 2016). The E_VB_ of BiOCl and BiOI were 3.60 eV and 2.11 eV, respectively. And the E_CB_ of BiOCl and BiOI were 0.26 and 0.37 eV, respectively.

**Figure 10:**
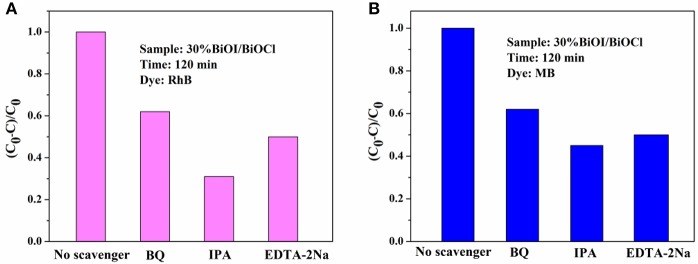
Trapping experiments of active species during visible light photodegradation: **(A)** RhB and **(B)** MB.

**Figure 11:**
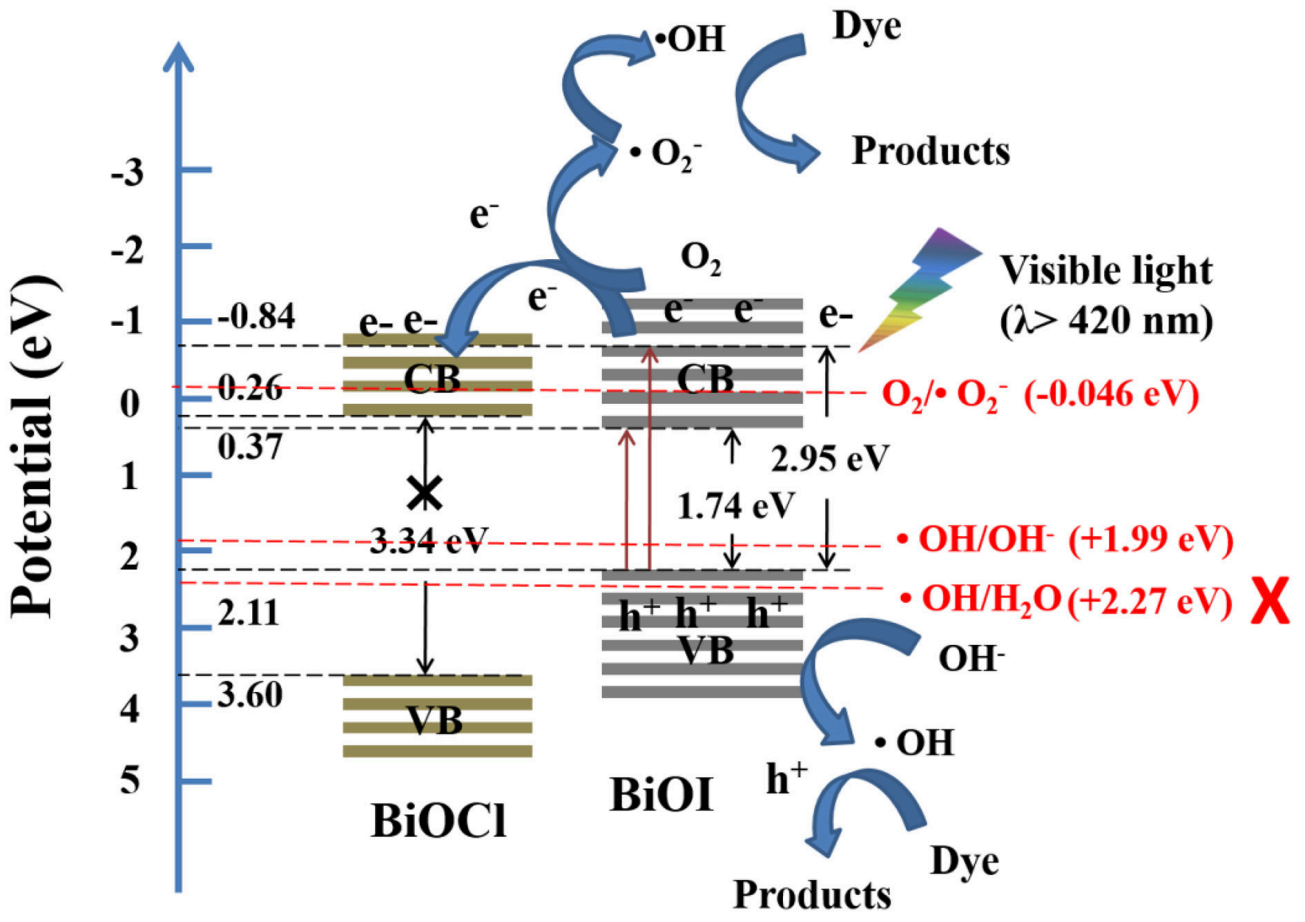
The proposed photodegradation mechanism of RhB and MB by BiOI/BiOCl films.

Active species of 30%BiOI/BiOCl film was detected by typical trapping experiments. Benzoquinone (BQ) was used as superoxide radical species (•${\text{O}}_{2}^{-}$) scavenger, while dimethylcarbinol (IPA) was used as quencher of •OH and EDTA-2Na was used as hole scavenger (h^+^). In Figures 10A,B, IPA could significantly decrease the photocatalytic efficiency; otherwise, BQ and EDTA-2Na had less effect on it. Figure 10 indicated that •OH, •${\text{O}}_{2}^{-}$ and h^+^ were active species during the degradation of RhB and MB. A possible mechanism of BiOI/BiOCl film was proposed based on the above discussion. In Figure 11, BiOI could utilize visible-light with energy < 2.95 eV (λ > 420 nm). Photoinduced electrons could be excited to a higher potential edge of BiOI (−0.84 eV) which was negative than that of BiOCl (0.26 eV). Then, photogenerated electrons could transfer to the CB of BiOCl, leaving the holes on the VB of BiOI. Thus, photogenerated carriers could be effectively separated. The E_VB_ of BiOI (2.11 eV) was negative than the potential of ·OH/H_2_O (2.27 eV), so •OH was generated by OH^−^ (E_•*OH*/*OH*^−^_ = 1.99 eV) rather than H_2_O (Zeng et al., 2016). Compared to the potentials of O_2_/•${\text{O}}_{2}^{-}$ (−0.046 eV), electrons in the BiOI/BiOCl could reduce O_2_ to •${\text{O}}_{2}^{-}$, followed by the generation of •OH (Wang et al., 2013; Zeng et al., 2016). In this way, h^+^, •${\text{O}}_{2}^{-}$ and •OH oxidized the organic compounds. which played an important role in the degradation process.

## Conclusions

In conclusion, BiOI/BiOCl films were successfully prepared using a facile method at room temperature. The growth process studies indicated that there was a hydrolyzation competition between BiOCl and BiOI in synthesis protocol. 30%BiOI/BiOCl could eliminate more than 99% of RhB within 90 min, which was 12 times higher than that of BiOCl. Besides RhB, 30% BiOI/BiOCl also showed a great photocatalytic performance toward MB. When degrading RhB, the efficiency of 30% BiOI/BiOCl was 5 and 12 times higher than that of pristine BiOI and BiOCl respectively. While degrading MB, 30%BiOI/BiOCl showed 6 times higher efficiency than that of pristine BiOI. These excellent enhancements were attributed to extended visible light region and high separation efficiency of charge. Five recycles indicated the as-prepared film exhibited a great reusability. In general, this work provided not only an easy and facial method to gain BiOI/BiOCl film but also insights for preparing photocatalysts which effectively utilized visible light with excellent reusability.

## Author contributions

YmZ, YZ, and HC participated in the design of this study; XY provided assistance for SEM, TEM, and data analysis; YxZ and YL carried out the experiments and drafted the manuscript. All the authors read and prove the final manuscript.

### Conflict of interest statement

The authors declare that the research was conducted in the absence of any commercial or financial relationships that could be construed as a potential conflict of interest.

## Supplementary material

Figure S1 XRD patterns of 30% BiOI/BiOCl films: fresh and after 5 cycles.

Figure S2 FT-IR spectra of samples: BiOCl, 30% BiOI/BiOCl, BiOCl.
